# Cytokine/chemokine patterns connect host and viral characteristics with clinics during chronic hepatitis C

**DOI:** 10.1186/2047-783X-17-9

**Published:** 2012-05-11

**Authors:** Antonios Katsounas, Martin Trippler, Shyam Kottilil, Richard A Lempicki, Guido Gerken, Joerg F Schlaak

**Affiliations:** 1 Department of Gastroenterology and Hepatology, University Hospital of Essen, Hufelandstr. 55, 45122, Essen, Germany; 2 Laboratory of Immunopathogenesis and Bioinformatics, SAIC-Frederick, Inc., NCI-Frederick, Frederick, MD, 21702, USA; 3 Laboratory of Immunoregulation, National Institute of Allergy and Infectious Diseases, National Institutes of Health, Bethesda, MD, 20892, USA

**Keywords:** HCV, Cytokines, Chemokines, Microarrays

## Abstract

**Background:**

In chronic hepatitis C virus (HCV) infection, liver tissue pathology and HCV genotype are important determinants of clinical and/or treatment-related outcome. Although consistent epidemiological and/or molecular-biological clues derived from different studies on single virus-host interactions are meanwhile published, the *in vivo* transcriptional responses and cellular pathways affected in >1 key aspects of the disease or treatment process are far from being understood.

**Methods:**

Microarray analysis was performed in peripheral whole blood (PB) samples from 36 therapy-naïve HCV-infected patients with known liver histology. Linear regression analysis identified gene expression profiles significantly correlating (*P* < 0.015) with ≥1 out of 7 variables: sustained viral response (SVR), viral non-response (NR), end of treatment viral response (ETR), viral breakthrough (VB), HCV genotype (Gt. 1 *vs.* Gt. 2/3), stage of hepatic fibrosis [St. 0/1 *vs.* St. 2/3/4] and grade of hepatic inflammation (Gr. 0/1 *vs.* Gr. 2/3/4). Correlation values across all seven contrasts were considered for hierarchical clustering (HCL).

**Results:**

A total of 1,697 genes showed ≥1 significant correlation results and genes involved in cell differentiation (183), immune response (53), and apoptosis (170) were leading fractions. HCL grouped the genes into six major clusters. Functional annotation analysis using DAVID (http://david.abcc.ncifcrf.gov) revealed that expression profiles that best linked these variables were highly enriched in cytokine/chemokine activity (Fisher-exact *P* < 0.0001) and specific biological module-centric algorithms finally led our focus on four out of fifty-three immune response genes: SMAD family member 3 (SMAD3), interleukin 1 receptor accessory protein (IL1RAP), tumor necrosis factor receptor superfamily member 1A (TNFRSF1A), and chemokine ‘C-C motif’ receptor 5 (CCR5). Of those, TNFRSF1A and CCR5 showed significant correlation with two out of seven variables based on microarray and/or quantitative real-time polymerase chain reaction (qRT-PCR) data.

**Conclusion:**

We identified molecular targets of the innate and adaptive immune system and validated their transcriptional specificity *in vivo* suggesting significant involvement in two unique outcomes during HCV treatment.

## Background

Hepatitis C virus (HCV) is estimated to persistently infect about 170 million people worldwide [1]. Histological grade of hepatic inflammation and stage of hepatic fibrosis as well as HCV genotype (Gt.) are considered significant determinants of clinical outcome in terms of progression of liver disease, and/or response to treatment in patients with chronic HCV infection (cHCV) as: (1) persistent he-patic inflammation is considered a major determinant of liver fibrosis progression towards cirrhosis [2]; (2) previous research has detected an association between advanced hepatic fibrosis and lower rates of successful response (sustained viral response (SVR)) to Interferon-alpha [3] or pegylated Interferon-alpha and Ribavirin (Peg-IFN-α/RBV) [4]; and (3) patients infected with Gt.2 or Gt.3 (80%) regularly achieve higher SVR rates relative to those infected with Gt.1 or Gt.4 (50%) [5]. To date, molecular targets/pathways significantly involved in multiple disease and/or treatment response determining host interactions with HCV have not been systematically studied or characterized. In this study, we analyzed gene expression profiles in peripheral whole blood (PB) of therapy-naïve HCV-infected subjects that underwent liver biopsy in order to identify gene expression patterns reflective of host biology that is linked to ≥1 clinical and/or viral stratification clusters. To this end, rigorous multicontrast linear regression analysis was performed on gene expression profiles against seven variables reflecting the state of cHCV with or without therapy. Our final results demonstrated that tumor necrosis factor receptor superfamily member 1A (TNFRSF1A) and chemokine ‘C-C motif’ receptor 5 (CCR5) correlated significantly with two out of seven variables. These findings, which have also been successfully validated by quantitative real-time polymerase chain reaction (qRT-PCR) in a fraction of patients, represent a promising basis for future molecular research aimed to provide comprehensive insights into genes/pathways with multiple *in vivo* impacts in viral hepatitis and/or develop anti-HCV therapies that target these genes/pathways.

## Methods

### Study subjects

Therapy-naïve Caucasian patients with cHCV (*n* = 36) received Peg-IFN-α2a (Pegasys®/Roche: 180 μg/week) or Peg-IFN-α2b (PEG-Intron®/Schering-Plough: 1.5 μg/kg/week) and Ribavirin (RBV, Rebetol®/Schering-Plough, 1000–1200 mg/d) for 48 weeks (Gt.1) or 24 weeks (Gt.3) and were followed up for 24 weeks post treatment at the University Hospital in Essen, Germany. All donors signed informed consents approved by the local board for ethics.

### Liver biopsy

Liver biopsies were obtained from all 36 patients prior to enrollment as part of their standard clinical assessments. All 36 specimens were evaluated using the Batts-Ludwig Grading (BL-G) and Staging (BL-S) system for viral hepatitis [6].

### Laboratory studies

Quantitative HCV RNA was performed 12 h before administration of the first dose of Peg-IFN-α and later during each study visit. Liver chemistry and safety laboratory tests were performed 12 h prior to treatment initiation and during each study visit.

### Microarray analysis

RNA (PAXgene-Blood-RNA-Kit, Qiagen, Hilden, Germany) isolated from PB was collected 12 h before the first injection of Peg-IFN-α directly into PAXgene-Blood-RNA-Tubes (Qiagen, Hilden, Germany). Total RNA, cRNA synthesis, and labeling along with hybridization to the U133A_2 or U133A arrays (Affymetrix, St Clara, CA, USA) were performed according to the manufacturer’s protocol. Gene expression values (log2) were determined using a correction for probe GC content, RMA background subtraction, and Quantile normalization. Chip-type-related batch effect was removed by regression analysis (PARTEK Genomics Suite).

Thirty-six patients were assigned to seven unique categories based on the criteria shown in Table 1: therapy outcome (SVR; NR, viral non-response; VB, viral breakthrough; ETR, viral relapse or end of treatment viral response), HCV genotype (Gt. 1 *vs.* Gt. 2/3), histological stage of hepatic fibrosis (St. 0/1 vs. St. 2/3/4), and histological grade of hepatic inflammation (Gr. 0/1 *vs.* Gr. 2/3/4). In a first step, gene expression profiles in PB were subjected to linear regression analysis against the abovementioned seven categorical variables. Then, expression profiles of genes that passed the strict statistical cutoff of *P* < 0.015 in at least one contrast were selected and further subjected to unsupervised hierarchical clustering (HCL) based on calculated linear regression values across all seven contrasts. Furthermore, functional annotation analysis using DAVID [7] was performed to identify biological activity that these expression profiles were most highly enriched in. Specific biological module-centric algorithms finally led our focus on four genes significantly involved in ‘immune response’. In a second step, we selected two out of those four genes by filtering the respective microarray data based on which expression profiles had reached statistical significance in at least one additional contrast. These results were validated by PCR in a fraction of patients (*n* = 9), of which RNA samples were currently available. 

**Table 1:** Distribution of patients across seven categorical disease-/therapy-related outcomes

**Symbol**	**Categorical title**	**Count (*n*)**
SVR	Sustained viral response	16/36
ETR	End of treatment viral response	7/36
VB	Viral breakthrough	5/36
NR	Viral non-response	8/36
Gt. 2,3	HCV genotype 2 or 3	6/36
Fibrosis St. 0,1	Histological stage of hepatic Fibrosis 0 or 1	21/36
	(Batts-Ludwig classification)	
Inflammation Gr. 0,1	Histological grade of hepatic Inflammation 0 or 1	23/36
	(Batts-Ludwig classification)	

Table 1 gives the numerical distribution of patients (*n* = 36), whose samples were subjected to microarray analysis at baseline, among seven groups representing categorical variables as defined under the Methods section.

Details on gene selection algorithms and clustering along with statistical degree and magnitude of associations are provided under the Results section. Correlation heat maps as well as clustering of microarray data have been performed using PARTEK Genomics Suite.

### qRT-PCR

Real-time detection of target gene messenger RNAs (mRNAs) with one-step quantitative PCR was performed on the Rotor-Gene 2000 real-time amplification system (Corbett Research). One-step qRT-PCR was carried out with the QuantiTect SYBR Green RT-PCR Kit (Qiagen) according to the manufacturer’s instructions. For the mRNA population per 1 gene, copy numbers were normalized to the number of beta-actin (β-actin) transcripts. Furthermore, the TNFRSF1A and CCR5 gene transcripts were quantified by qRT-PCR using specific QuantiTect Primer Assays (Qiagen).

### Statistical analysis

Linear regression analysis was performed using one-way ANOVA. Statistics and Graphics were performed in PARTEK Genomics Suite. The significance cutoff was chosen not to exceed 5% (*P* < 0.05). As shown elsewhere [8], for analysis checking two contrasts at once we relaxed *P* to ≤0.1 only for one contrast in order not to risk missing significant biology and obtain a slightly larger gene set for validation qRT-PCR.

## Results

**Figure 1:**
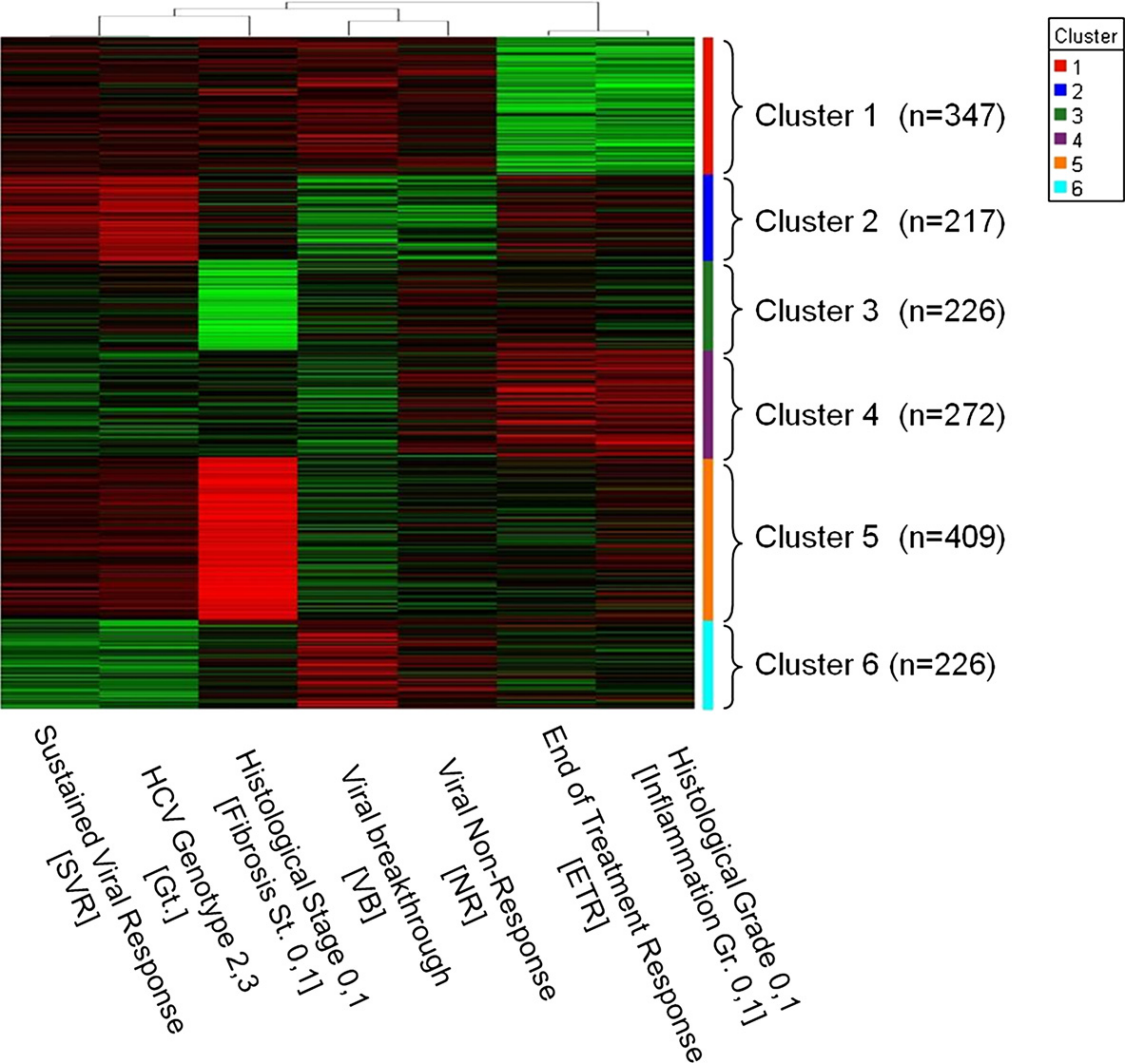
**Unsupervised (hierarchical) clustering grouped 1,697 genes significantly correlating with ≥1 of the displayed seven categorical variables (*P*** **< 0.015) into six major clusters.** Red color reflects (+) correlation; green color reflects (−) correlation.

In this study, we analyzed expression patterns of genes significantly correlating with one or more HCV-related parameters, liver disease outcomes and/or responses to anti-HCV treatment. More specifically, gene expression profiles in PB of therapy-naïve patients with cHCV were subjected to linear regression analysis against seven categorical variables shown in Table 1. Gene expression profiles (*n* = 1,697) that reached at least one highly significant correlation result (r > 0.4 or r < −0.4, *P* < 0.015) were selected and further subjected to unsupervised hierarchical clustering (HCL) using linear regression values calculated across all seven contrasts (Figure 1). HCL grouped the genes into six major clusters providing a detailed view of the relationship between gene expression profiles, clinic- or therapy-related outcomes and viral characteristics. Genes involved in immune response (*n* = 53), cell differentiation (*n* = 183), and apoptosis (*n* = 170) were leading fractions across these clusters based on functional annotation analysis using DAVID [7]. Expression profiles that best linked these parameters were highly enriched in ‘cytokine/chemokine activity’ (DAVID [7], Fisher exact *P* < 0.0001). 

Then, we focused on four out of the fifty-three genes involved in ‘immune response’ through specific biological module-centric algorithms provided by DAVID [7], that is SMAD family member 3 (SMAD3), interleukin 1 receptor accessory protein (IL1RAP), TNFRSF1A, and CCR5. Among those, CCR5 gene expression (microarray) showed a strong positive correlation with SVR (r = 0.29, *P* = 0.08) and an inverse correlation with VB (r = −0.38, *P* = 0.02, Additional file 1: Table S1) at baseline. TNFRSF1A gene expression (microarray) exhibited positive correlation with SVR (r = 0.41, *P* = 0.01) and an inverse correlation with NR (r = −0.35, *P* = 0.03, Additional file 1: Table S1) at baseline. In line with these findings were the results from the exact same type of analysis including gene expression data generated by qRT-PCR. Thus, CCR5 gene expression (qRT-PCR) showed a positive correlation with SVR (r = 0.78, *P* = 0.01) and an inverse correlation with VB (r = −0.58, *P* = 0.09, Additional file 2: Table S2) at baseline. TNFRSF1A gene expression (qRT-PCR) exhibited a positive correlation with SVR (r = 0.64, *P* = 0.06) and an inverse correlation with NR (r = −0.57, *P* = 0.1, Additional file 2: Table S2) at baseline. Since only a small count of samples were available for qRT-PCR (*n* = 9), caution should be exerted when interpreting the *P*; therefore, mainly correlation values should be used as comparable measures between microarray and qRT-PCR results.

Knowing this, we should also note that qRT-PCR data often showed a lower *P* relative to microarray data due to the higher correlation values calculated by one-way ANOVA in all contrasts. However, results obtained for TNFRSF1A and CCR5 on the level of both microarray and qRT-PCR analyses are statistically tight. Consequently, our approach considering these genes important players for multiple *in vivo* outcomes (despite some associations with the abovementioned variables at a relaxed *P* of ≤0.1 [8] in different settings, i.e. microarray *vs*. qRT-PCR) likely identified active and meaningful biological relationships. Results for SMAD3 reached significance in no contrast based on qRT-PCR expression values and showed predominantly inconsistent trends relative to microarray data (Additional file 1: Tables S1 and Additional file 2: Table S2). Interestingly, IL1RAP showed only on the level of qRT-PCR data analysis a positive correlation with SVR (r = 0.75, *P* = 0.01).

Taken together, this study identified molecular markers/pathways of the innate and adaptive immune system that potentially exert multifaceted *in vivo* roles in cHCV as indicated by statistical correlations between two unique disease-related outcomes and gene expression profiles detected by microarray analysis and validated by qRT-PCR.

## Discussion

The present study was aimed to identify human genes/pathways participating in multiple host interactions with HCV *in vivo*. The analytical design chosen provided a unique opportunity to use the microarray-technique as a molecular readout for the differential regulation of genes/pathways with diverse potential implications in cHCV. Thus, pattern analysis of microarray data based on gene expression significantly correlating with one or more viral characteristics, histological liver pathologies, and/or final treatment responses led to identification of specific gene clusters strongly associated with the cytokine/chemokine activity *in vivo*. These observations appear biologically consistent with previous research linking liver disease and treatment outcome during cHCV to responses of both the innate and adaptive immune system orchestrated by networks of cytokines and chemokines [9,10]. Particularly, various cytokines and chemokines are reportedly involved in affecting host susceptibility to developing chronic hepatitis C or response of cHCV to IFN-α therapy [11,12].

Using microarray analysis, combined with functional annotation tools such as DAVID [7], we detected specific changes in gene expression of four molecular markers assigned to the functional annotation category ‘immune response’, that is SMAD3, IL1RAP, TNFRSF1A, and CCR5, which exhibited significant correlation with two categorical variables representing unique therapy outcomes. Validation qRT-PCR confirmed these results for TNFRSF1A and CCR5. In contrast, linear regression analysis using qRT-PCR data for SMAD3 did not detect any significance; therefore, SMAD3 has been omitted from the Discussion section. As specific interactions between HCV and SMAD3 have been previously identified but are, however, beyond this article’s scope, interested readers are referred for details to the respective original literature [13].

Moreover, increased TNFRSF1A gene expression at baseline was found in significant correlation with HCV elimination in terms of SVR and inverse relation to HCV persistence due to complete viral non-response (NR, that is non-ETR or non-VB). These observations are in agreement with previous publications showing up-regulation of TNFRSF1A expression in peripheral dendritic cells of HCV-infected patients who reach SVR [14].

In recent years, increasing amounts of evidence have indicated that spontaneous HCV clearance or successful response to antiviral treatment display a predominant T helper 1 (Th1) lymphocyte-related cytokine/chemokine profile including CCL5 and its specific receptor CCR5 [15]. In this light, the positive correlation between CCR5 gene expression levels and SVR as well as the inverse correlation between CCR5 gene expression and VB, which is the most immediate type of treatment failure, both observed in the present study, strongly suggest the good prognostic value along with the potentially favorable biological role of increased CCR5 expression in the peripheral blood at baseline.

There are several limitations of this study that warrant comment. First, patients in relative low numbers failed to clear HCV due to ETR (*n* = 7) or VB (*n* = 5), which may have affected the statistical power of the linear regression model and introduced errors in calculation of significance in ETR- and/or VB-based contrasts. Second, this study was able to provide confirmation of significant correlations against validated results via qRT-PCR in only 25% (9/36) of patients whose samples were originally subjected to microarray analysis. Third, protein expression has not been investigated for identified gene targets; thus, posttranscriptional expression and true level of functional importance of these genes remains obscure. Future work will address these concerns in ongoing clinical trials with larger numbers of HCV-infected subjects treated with Peg-IFN-α/RBV and/or new direct-acting antiviral agents.

## Conclusions

In conclusion, this study provides foundation for greater understanding of the multifaceted role of the immune system in the outcome of chronic hepatitis C by associating patient stratification based on standard clinical variables with biologically significant genes/pathways likely involved in numerous key aspects of the disease and treatment process *in vivo*.

## Abbreviations

ETR: End of treatment response or viral relapse (HCV-RNA not detectable at the end of antiviral treatment with a subsequent increase in serum HCV RNA levels before the end of follow-up); NR: Viral non-response (presence of HCV RNA in serum at the end of a minimum of 24 weeks (for Gt. 2/3) or 48 weeks (for Gt. 1/4) of therapy; SVR: Sustained viral response (HCV-RNA not detectable at the end of follow-up, that is week 24 after completion of antiviral treatment); VB: Viral breakthrough (an abrupt increase in serum HCV RNA levels after a period of persistent suppression before completion of antiviral treatment).

## Competing interests

The authors declare that they have no competing interests.

## Authors’ contribution

AK conducted study design, performed microarray analysis and drafted 90% of the manuscript. MT partially provided description of the methods section, performed validation experiments using qRT-PCR for the final target genes and completed normalization of expression values against the housekeeping gene “β-actin”. SK provided immunological and hepatological evaluation of selected results and performed valuable draft editing of the manuscript prior to first submission. RAL provided supervision of microarray analysis and statistics along with quality control of the functional annotation analysis and interpretation of results. GG provided supervision of clinical management of study patients along with control of clinical and histological data, received clearance from the ethics board to conduct this study and supervised drafting of the manuscript before and after first submission. JFS, as a senior author, led this study and provided scientific expertise and supervision of all stages of preparation of this manuscript before and after first submission. All authors read and approved the final manuscript.

## Disclosure

None of the authors has any commercial or other association that might pose a conflict of interest.

## Supplementary Material

Additional file 1: Table S1 Final linear regression analysis results based on microarray data at baseline [16]. Click here for file

Additional file 2: Table S2 Final linear regression analysis results based on qRT-PCR data at baseline [16]. Click here for file
