# Supplementary material for: Cytokine/chemokine patterns connect host and viral characteristics with clinics during chronic hepatitis C
Source: Eur J Med Res. 2012 May 11;17(1):9. doi: 10.1186/2047-783X-17-9 (PMC3489717; doi:10.1186/2047-783X-17-9)
Supplement: Additional file 1: Table S1 — Final linear regression analysis results based on microarray data at baseline [16]. [file 2047-783X-17-9-S1.doc]

**Additional file 1: Table S1. Final linear regression analysis results based on microarray data at baseline**

| **Variable** | **SVR** | **Gt 2,3** | **Fibrosis** **St. 0,1** | **VB** | **ETR** | **NR** | **Inflammation** **Gr. 0,1** | **Statistics** |
| --- | --- | --- | --- | --- | --- | --- | --- | --- |
| **CCR5** **(206991_s_at)** | 0.290555 | 0.321037 | 0.0612856 | -0.381594 | 0.291831 | -0.307668 | 0.0730266 | r |
| **IL1RAP** **(205227_at)** | 0.020001 | -0.130676 | 0.102963 | 0.212731 | -0.225958 | 0.0142418 | -0.18812 | r |
| **SMAD3** **(205396_at)** | 0.184644 | 0.151674 | -0.218653 | -0.223601 | -0.095483 | 0.0562069 | -0.0909428 | r |
| **TNFRSF1A** **(207643_s_at)** | 0.416855 | 0.0575538 | -0.156464 | -0.027607 | -0.13077 | -0.350782 | -0.155145 | r |
| **CCR5 (206991_s_at)** | 0.085593 | 0.0562463 | 0.722537 | **0.0216514** | 0.084166 | 0.0679374 | 0.672104 | *P* value |
| **IL1RAP (205227_at)** | 0.907829 | 0.447463 | 0.550129 | 0.21289 | 0.185133 | 0.934297 | 0.271885 | *P* value |
| **SMAD3 (205396_at)** | 0.281003 | 0.377208 | 0.200128 | 0.189882 | 0.579614 | 0.744729 | 0.59785 | *P* value |
| **TNFRSF1A (207643_s_at)** | **0.011433** | 0.738823 | 0.362146 | 0.873015 | 0.447133 | **0.0359419** | 0.366258 | *P* value |

Table 2 shows linear regression analysis results between gene expression of CCR5, IL1RAP, SMAD3, TNFRSF1A measured by microarray in patients with cHCV (*n* = 36) and seven categorical variables as defined under the Methods section. All associations at a relaxed *P* of ≤0.1 along with the respective correlation values (r) are displayed in black. Correlations at a *P* < 0.05 are highlighted (bold).

Table 2 and 3. Only correlation results calculated for CCR5 and TNFRSF1A both on the level of microarray and qRT-PCR analysis showed (at a relaxed *P* of ≤0.1) statistically and biologically consistent patterns (= grey cells). Results for SMAD3 did not reach any significance. Interestingly, IL1RAP showed only on the level of qRT-PCR a positive correlation with SVR. This finding is likely attributable to small number statistics or different splicing variants of the molecule [16] detected by microarray *vs.* qRT-PCR and has to be validated in larger cohorts.
