# Supplementary material for: Cytokine/chemokine patterns connect host and viral characteristics with clinics during chronic hepatitis C
Source: Eur J Med Res. 2012 May 11;17(1):9. doi: 10.1186/2047-783X-17-9 (PMC3489717; doi:10.1186/2047-783X-17-9)
Supplement: Additional file 2: Table S2 — Final linear regression analysis results based on qRT-PCR data at baseline [16]. [file 2047-783X-17-9-S2.doc]

**Additional file 2: Table S2. Final linear regression analysis results based on qRT-PCR data at baseline**

| **Variable** | **SVR** | **Gt 2,3** | **Fibrosis** **St. 0,1** | **VB** | **ETR** | **NR** | **Inflammation** **Gr. 0,1** | **Statistics** |
| --- | --- | --- | --- | --- | --- | --- | --- | --- |
| **CCR5 (sign/ßact)** | 0.786659 | -0.255793 | 0.160463 | -0.58802 | -0.440237 | -0.020162 | -0.013669 | r |
| **IL1RAP (sign/ßact)** | 0.751688 | 0.0156073 | -0.19992 | -0.416928 | -0.268669 | -0.066502 | 0.176884 | r |
| **SMAD3 (sign/ßact)** | 0.285034 | -0.419899 | -0.17423 | -0.062274 | -0.113095 | -0.165504 | 0.400548 | r |
| **TNFRSF1A (sign/ßact)** | 0.644684 | 0.164071 | -0.337599 | -0.164766 | -0.014562 | -0.577756 | -0.157261 | r |
| **CCR5 (sign/ßact)** | **0.011901** | 0.506486 | 0.680041 | 0.0958455 | 0.235668 | 0.958938 | 0.972158 | *P*-value |
| **IL1RAP (sign/ßact)** | **0.019509** | 0.968212 | 0.606048 | 0.264256 | 0.484535 | 0.865025 | 0.648918 | *P* value |
| **SMAD3 (sign/ßact)** | 0.457223 | 0.260515 | 0.653918 | 0.873544 | 0.772046 | 0.67044 | 0.285384 | *P* value |
| **TNFRSF1A (sign/ßact)** | 0.060858 | 0.673165 | 0.374266 | 0.671843 | 0.970339 | 0.100002 | 0.68616 | *P* value |

Table 2 shows linear regression analysis results between gene expression of CCR5, IL1RAP, SMAD3, TNFRSF1A measured by qRT-PCR in patients with cHCV (*n* = 9) and seven categorical variables as defined under the Methods section. All associations at a relaxed *P* of ≤0.1 along with the respective correlation values (r) are displayed in black. Correlations at a *P* < 0.05 are highlighted (bold).

Only correlation results calculated for CCR5 and TNFRSF1A both on the level of microarray and qRT-PCR analysis showed (at a relaxed *P* of ≤0.1) statistically and biologically consistent patterns (= grey cells). Results for SMAD3 did not reach any significance. Interestingly, IL1RAP showed only on the level of qRT-PCR a positive correlation with SVR. This finding is likely attributable to small number statistics or different splicing variants of the molecule [16] detected by microarray *vs.* qRT-PCR and has to be validated in larger cohorts
